# Meteorological Table

**Published:** 1812-01

**Authors:** 


### METEOROLOGICAL TABLE.

*From November 26, to December 26.*

| D. | Therm. | | | Barom. | | Hygrom. dry——damp | | | Winds. | Atmos. Variation. |
|---|---|---|---|---|---|---|---|---|---|---|
| 27 | 44 | 45 | — | $30^{4}$ | — | 22 | — | — | W . | C ... — .. F . |
| 28 | 44 | 47 | 45 | $30^{3}$ | $^{2}$ | 22 | — | — | NW . | C .... — .... — .... |
| 29 | 44 | 47 | — | $30^{3}$ | $^{2}$ | 24 | 23 | — | NW . | C ... F .. C ... |
| ○ 30 | 47 | 50 | 48 | $30^{2}$ | — | 24 | 22 | — | SW . | C .... F .. C ... |
| 1 | 46 | 50 | — | $30^{1}$ | $29^{8}$ | 23 | — | 19 | S . . | F .. R .. — ... |
| 2 | 50 | 45 | 39 | $29^{5}$ | — | 26 | — | 20 | W . . . | R ... C .. F ... |
| 3 | 43 | 46 | 49 | $29^{4}$ | $^{5}$ | 25 | — | 27 | W . . . | C ... F ... — ... |
| 4 | 42 | 44 | 36 | $29^{3}$ | — | 22 | 20 | 22 | W . . . | F..—...—Snow in N. |
| 5 | 35 | 32 | 29 | $29^{4}$ | $^{0}$ | 19 | 15 | 11 | N . . . | F ... — ....: — .... |
| 6 | 29 | 36 | 39 | $29^{9}$ | — | 19 | — | 20 | N . W .. | F ... Fog .. R .. |
| ◐ 7 | 47 | 50 | — | $29^{7}$ | $^{5}$ | 30 | 35 | 38 | SW . . | C.... R .. — ... in N. |
| 8 | 52 | 53 | 46 | $29^{5}$ | $^{4}$ | 40 | 35 | 30 | S . . . | C.... R .. — ... in N. |
| 9 | 44 | 43 | 42 | $29^{3}$ | $^{1}$ | 30 | — | 36 | S . . . | C....R..F..R... in N. |
| 10 | 43 | — | 40 | $28^{9}$ | $29^{4}$ | 30 | — | — | NW . . | C .... F ... — .. . |
| 11 | 41 | 43 | 40 | $29^{6}$ | $^{9}$ | 30 | 24 | 30 | NE . | C .... F .. C ... |
| 12 | 36 | 42 | 47 | $29^{9}$ | $^{8}$ | 30 | — | 34 | NW .. W ... | C .. — .... R ... |
| 13 | 51 | 54 | 50 | $29^{8}$ | $^{6}$ | 38 | 35 | 30 | W . . | C..... — ... R ... |
| 14 | 40 | 43 | 38 | $29^{8}$ | 30 | 30 | 19 | 30 | N . . | F ... — ... — ... |
| ● 15 | 37 | 43 | 45 | 30 | $29^{6}$ | 27 | 24 | 36 | SW . | F ... C .. R .. in N. |
| 16 | 39 | 41 | — | $29^{4}$ | $^{3}$ | 28 | 21 | 26 | W . . | F ... — ... R .. |
| 17 | 40 | — | 39 | $29^{6}$ | $^{7}$ | 25 | — | 20 | NW . . | C .... — ... R .. |
| 18 | 37 | 48 | 47 | $29^{8}$ | — | 30 | 25 | 33 | W . . | F .. R .. C .... |
| 19 | 50 | 51 | 49 | $29^{7}$ | — | 40 | 30 | 37 | SW . . | C .... — .. R .. |
| 20 | 51 | 52 | 50 | $29^{7}$ | $^{6}$ | 38 | 36 | 38 | S . . | R .. — .. — .. |
| 21 | 50 | 47 | 37 | $29^{5}$ | 30 | 32 | 30 | 35 | W . . E . . | C .... R .. F ... |
| ◑ 22 | 32 | 36 | 35 | $30^{1}$ | — | 26 | 20 | 27 | N . . | F ... — .... — .... |
| 23 | 43 | 47 | 44 | 30 | — | 30 | — | 23 | W . . | C .... F ... — .... |
| 24 | 43 | 44 | 42 | $30^{1}$ | 30 | 29 | — | 25 | N . | C .... R . C ... |
| 25 | 35 | 38 | 33 | $30^{2}$ | — | 22 | 20 | 17 | N . | F .... — .... — ....; |
| 26 | 32 | 35 | 34 | $29^{9}$ | $^{7}$ | 24 | — | 20 | NE . | Fog ... Snow .. F .... |

The quantity of rain from November the 26th to December the 26th, 1 inch $\frac{85}{100}$.

First snow on the 4th of December, which dissolved in a few hours. On the 26th snow again, covering the streets and houses, and remaining on the morning of the 27th. Though the range of temperature in this month has been very great, from 54 to 29, the weather was pleasant for the season; the 5th and the 22d were brilliant days. The quantity of rain has not been much above one-third of what fell from October the 26th to November the 26th.

The lowest degree to which the thermometer has fallen in 1811, was 27° on the 5th of January, wind E... and the day fair. The highest point to which it has risen, was 81° on the 23th of July at noon, fair and wind S. On the morning of this day the thermometer 66, wind E.

The quantity of rain from the 28th of March, when the register began, to the 26th of December, is 17 inches $\frac{63}{100}$. From the 26th of October, to the 26th of November, was the wettest interval, 3 inches $\frac{33}{100}$ of rain falling; the next wettest was from the 26th of June to the 26th of July, in which 3 inches $\frac{27}{100}$ fell.

As the range of the thermometer in this register may possibly vary from registers kept in other places, it may be proper to observe, that the instrument here used constantly hangs in one place in the open air, is neither exposed to the direct nor reflected rays of the sun, nor to currents of wind.

*Prince's-street, Cavendish square.*

